# Surface Enhanced Raman Scattering (SERS) Studies of Gold and Silver Nanoparticles Prepared by Laser Ablation

**DOI:** 10.3390/nano3010158

**Published:** 2013-03-01

**Authors:** Gloria M. Herrera, Amira C. Padilla, Samuel P. Hernandez-Rivera

**Affiliations:** ALERT-DHS Center of Excellence/Center for Chemical Sensors Development, University of Puerto Rico-Mayagüez, P.O. Box 9000, Mayaguez, PR 00681-9000, USA; E-Mails: gloriam.herrera@upr.edu (G.M.H.); amirac.padilla@upr.edu (A.C.P.)

**Keywords:** laser ablation, Au and Ag NP, Raman spectroscopy, SERS, explosives

## Abstract

Gold and silver nanoparticles (NPs) were prepared in water, acetonitrile and isopropanol by laser ablation methodologies. The average characteristic (longer) size of the NPs obtained ranged from 3 to 70 nm. 4-Aminobenzebethiol (4-ABT) was chosen as the surface enhanced Raman scattering (SERS) probe molecule to determine the optimum irradiation time and the pH of aqueous synthesis of the laser ablation-based synthesis of metallic NPs. The synthesized NPs were used to evaluate their capacity as substrates for developing more analytical applications based on SERS measurements. A highly energetic material, TNT, was used as the target compound in the SERS experiments. The Raman spectra were measured with a Raman microspectrometer. The results demonstrate that gold and silver NP substrates fabricated by the methods developed show promising results for SERS-based studies and could lead to the development of micro sensors.

## 1. Introduction

The many uses of nanotechnology include very important applications in areas, such as medicine [1,2,3,4,5], catalysis [6,7,8,9,10,11,12,13,14], industrial applications [15,16,17] and scientific investigations. The size, shape and physicochemical properties are very important in future applications and are the main theme in studies currently conducted. There are several ways to synthesize metal nanoparticles (NPs). Their synthesis can be classified as either chemical or physical methods. Some chemical methods include the chemical reduction of metal salts, the alcohol reduction process, the polyol process, microemulsions, the thermal decomposition of metal salts and electrochemical synthesis [18,19]. Physical methods include pulsed laser ablation, the exploding wire technique, plasma, chemical vapor deposition, microwave irradiation, supercritical fluids, sono-chemical reduction and gamma radiation [20]. Laser ablation is a very clean physical method for the preparation of metallic nanoparticles (NPs). During the last year, the laser ablation of metals has increased in popularity, due to the fast and simple nature of the procedure. In addition, the important advantage of this method when compared to chemical synthesis (“wet chemistry syntheses”) is the preparation of high surface purity NPs in the chosen solvent. There are no counter ions and no residuals of the reducing agents remaining on the surfaces of the NPs [21,22,23]. To take advantage of these features, we have prepared gold (Au) and silver (Ag) NPs by laser ablation. Colloidal suspensions of prepared NPs were deposited on gold-coated slides to immobilize them and to test them for potential use as substrates for the detection of explosives using surface enhanced Raman scattering (SERS).

The use of spectroscopic techniques with intensities augmented by nanostructured metal surfaces has attracted great interest in recent years. The SERS effect discovered in the seventies is largely attributed to the interaction of light with matter. Specifically, SERS is related to the inelastic scattering (or Raman scattering) of certain molecules in the presence of specially prepared roughened or discontinuous metallic nanostructures. There are two mechanisms that explain the increase in the Raman signal. The first is explained through an electromagnetic interaction model (EM) and the second through a chemical interaction model or charge transfer (CT) [24,25]. Both mechanisms are thought to contribute to the signal intensity enhancement observed, although the extent of the contribution of each source of enhancement depends on the system under study. The enhancement mechanisms of the Raman signal lead to a technique with a sensitivity and selectivity that make Raman scattering a highly promising technique for further developing analytical applications [26,27,28,29,30,31]. These applications are closely related to the properties and the surface morphology of the metallic NP used. A large part of the contribution to the SERS effect is due to the increase in the inelastically scattered Raman signal intensity by the nanostructured metal systems present and the particular properties of the particles to induce greater morphological coupling with the incident radiation, resulting in intense spectroscopic signals. The molecule-surface relative orientation allows the emergence of new selection rules, resulting in the intensification of the Raman spectrum bands corresponding to the molecular vibrations of the molecular polarizability components perpendicular to the surface. The Au and Ag NPs prepared by laser ablation were deposited on various substrates and subsequently evaluated as SERS substrates, with the objective of detecting explosives, such as TNT.

## 2. Experimental Section

### 2.1. Laser Ablation Synthesis

Au or Ag metal foils (99.99%, Sigma-Aldrich, Milwaukee, WI, USA) were placed in a vial containing 10 mL of deionized water as ablation and heat tempering media. Laser pulses at 1064 nm, obtained using a Quanta-Ray Pro Series pulsed Nd:YAG laser from Spectra-Physics/Newport Corporation (Mountain View, CA, USA), were used to ablate the metallic foils. The laser was operated in single-shot mode (5 ns, 10 Hz). The target was irradiated using a focusing lens with a focal length of 86.4 cm. The laser power used was 0.980 mW, and the energy was 106 mJ. The ablation process was carried out for time intervals of 5, 10, 15 and 20 min of near IR laser pulse irradiation.

### 2.2. Characterization of NPs Suspensions

A UV-Vis spectrophotometer (Agilent model 8453, Santa Clara, CA, USA) was used to acquire the electronic absorption spectra of the NPs in water. The spectra were recorded in the range of 300 to 900 nm. Quartz cells with a 1.0 cm path length (72-Q-10, obtained from Starna Cells, Inc., Atascadero, CA, USA) were used for the experiments. The NPs morphology and size were obtained from high-resolution transmission electron microscopy (HR-TEM) images (Zeiss, model 922 operated at 200 kV). The samples for TEM analysis were prepared by depositing 5 μL of the metallic NP suspensions on ultrathin carbon film/holey carbon 400 mesh copper grids (01824 from Ted Pella, Inc., Redding, CA, USA). Zeta potential and hydrodynamic radius (HR) measurements were obtained using a Zetasizer™ Nano Series (Malvern Instruments Ltd., Worcestershire, UK).

### 2.3. Effect of pH on the Synthesis of NPs

Solutions of 1 mM 4-ABT were used as analytes to evaluate the pH effect on the synthesis by laser ablation of colloidal suspensions of Au and Ag NPs. The preparation method used was as described in Section 2.1. Studies were performed only at the optimum irradiation time. Dilute solutions of NaOH and HCl were used to adjust the pH in the aqueous media used as the solvent in the synthesis of nanoparticles. After the synthesis at various pH values was studied, the UV-Vis spectra of the suspensions were obtained. The pH values of the aqueous colloidal suspensions used for synthesis were 2.6, 4.8, 8.1 and 10.3.

### 2.4. Evaluation of SERS Activity

SERS spectra were excited with a 514.5 nm INNOVA 308 Argon ion laser or a 532 nm VERDI 6.0 solid-state diode laser (both from Coherent, Inc., Santa Clara, CA, USA) and a 785 nm solid-state laser (InProcess Inc., Salt Lake City, UT, USA). 4-Aminobenzebethiol (4-ABT, Sigma-Aldrich) and 1,2-bis(4-pyridyl)ethylene (BPE, Sigma-Aldrich) were used as SERS probe analytes. Renishaw Raman Microspectrometers RM1000 and RM2000 systems (Agiltron, Inc., Woburn, MA, USA) were used to acquire normal Raman (NR) and SERS spectra. The laser power at the samples was typically in the range of 10–60 mW. The data acquisition time was 20 s with 2 accumulations. The spectra are presented without pre-treatments or baseline corrections.

Au and Ag NPs at different pH values were used to evaluate the effect of the pH of the colloidal suspensions on the SERS activity obtained. Thus, 1.0 mM 4-ABT solutions and TNT solutions were used to evaluate the SERS activity at different pH values. Aliquots of 3 μL of TNT solutions at 1.0 mM were deposited on Au NP/Au substrates. To determine the surface enhancement factor (SEF), a solution of 4-ABT at 1.0 × 10^−9^ M was deposited on Au NP/Au substrates (approximately 5–10 μL of Au NPs deposited on Au substrates).

### 2.5. Detection of TNT Using Au NP/Au Substrates

The low limit of detection (LOD) of TNT using Au NPs deposited on Au-coated glass slides and used as substrates for the NPs was calculated based on the SERS data. For the spectral measurements, aqueous solutions of 2,4,6-trinitoluene (TNT) of 1.0 × 10^−4^ M, 1.0 × 10^−6^ M and 1.0 × 10^−10^ M were used. Aliquots of 10 μL of Au NPs were deposited on Au-coated substrates, and Raman spectra were measured. Then, 2.5 μL of TNT solutions were deposited on Au NP films. The NPs used were prepared in acetone to facilitate the evaporation of the solvent and the quicker use of the substrate.

## 3. Results and Discussion

**Figure 1:**
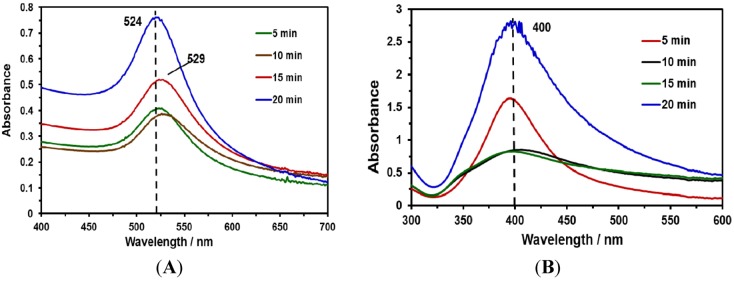
UV-Vis absorption spectra of Au and Ag nanoparticles (NPs) at various irradiation times: (**A**) absorption spectra of Au NPs; (**B**) absorption spectra of Ag NPs.

This contribution focuses on developing fast and simple methods for the preparation of SERS-active substrates with high sensitivity. For this purpose, NPs were synthesized using laser ablation methods. Au and Ag NP suspensions were synthesized at different irradiation times (5, 10, 15 and 20 min). UV-Vis absorption measurements were obtained to characterize the NPs obtained (Figure 1A,B). The typical positions of the surface plasmon maximum absorption wavelength for Au NPs and Ag NPs were approximately 525 nm (Figure 1A) and 400 nm (Figure 1B), respectively. These absorption maxima correspond to spherical (or nearly spherical) NPs with a characteristic average diameter between 2 and 100 nm [32]. These results were corroborated with TEM images and the corresponding statistical and morphological analyses.

**Figure 2:**
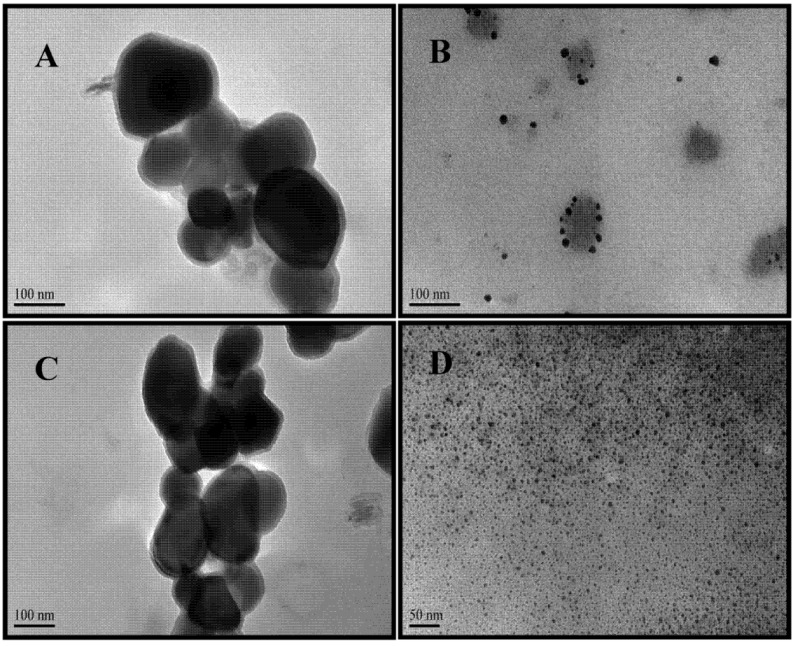
TEM images of Au and Ag NPs. Au NPs: (**A**) large spheres with average diameters of 126 ± 39 nm are violet. (**B**) red colloids have average diameters of 11 ± 4 nm Ag NPs: (**C**) yellow Ag NPs suspensions have average sizes of 132 ± 5 nm; (**D**) silver seed-like NP with an average long axis of 5 ± 1 nm are green-gray.

TEM images of the prepared Au and Ag NPs are shown in Figure 2. Colloidal suspensions of Au NPs of different sizes were obtained. Purple colloids are typical of large Au NPs of approximately 126 ± 36 nm (Figure 2A). Red colloid suspensions have average sizes of 11 ± 4 nm (Figure 2A). The spherical shape is predominant in the images. Similarly, Figure 2C,D show TEM images of typical colloidal suspensions of Ag NPs. Green-gray colloidal suspensions have NPs with spheroidal, large Ag NPs of approximately 132 ± 5 nm and yellow Ag NPs are spherical seeds of 5 ± 1 nm. The average sizes of the NPs shown in these TEM images were determined using the “ImageJ” program (NIH).

**Figure 3:**
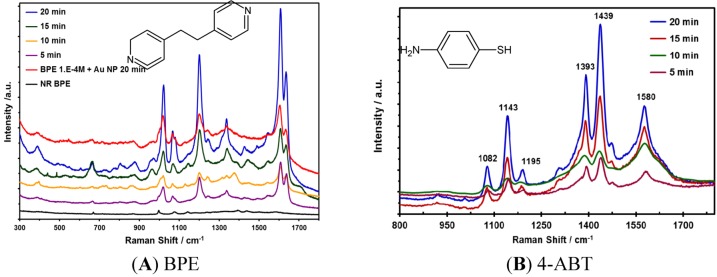
Surface enhanced Raman scattering (SERS) spectrum of (**A**) 1 mM BPE in Au NPs at various irradiation times. Raman and SERS spectra were acquired at 785 nm. (**B**) 1 mM 4-ABT deposited on Ag NPs deposited on Au-coated glass slide at various irradiation times. SERS spectra were acquired at 532 nm.

The effect of the irradiation time on the enhancements obtained in the SERS experiments using the metallic NPs prepared was evaluated using 4-ABT for Ag NPs. Similarly, 1,2-bis(4-pyridyl)ethylene (BPE) was used to evaluate the optimum conditions for the synthesis of Au NPs. These analytes were selected based on the affinity of the compounds with the corresponding NPs. Figure 3 displays the SERS spectrum results of Au NPs at different irradiation times. The best enhancement in the Raman signals for 4-ABT was observed at 20 min of irradiation time for Ag and Au NPs. The optimized parameter (20 min of irradiation) was used during all subsequent syntheses in this work. Aliquots of 5 μL of NP suspensions at different times of analysis were deposited on Au slides of approximately 0.25 cm^2^. The drops of NP suspensions were allowed to dry in a desiccator overnight, and then, the analyte was deposited on the substrate and again placed in a desiccator.

Predominant vibrational signals were observed in the Raman spectrum of 4-ABT at 1140, 1390 and 1430 cm^−1^. These signals can be attributed to modes assigned to the 9b, 3 and 19b modes of the b2-type ring, respectively. The low intensity band at 1080 cm^−1^ is due to the 7a mode of the a1-type ring [33,34,35,36,37]. The main Raman bands of BPE are observed in SERS spectrum of the probe molecules deposited on Au NPs. The peak at 994 cm^−1^ corresponds to the ring breathing mode of BPE pyridine. A blue shift of 27 cm^−1^ was observed in the SERS spectra of BPE, which includes the vibrational movement of the pyridyl nitrogen atom. Similarly, the vibrational signature observed at 1596 cm^−1^ corresponds to the C–N stretching mode of the pyridyl ring. This band presents a blue shift of approximately 10 cm^−1^ in the SERS spectrum of BPE deposited on Au NPs. These results suggest that the molecule of BPE interacts strongly with the surface of the Au NPs through the nitrogen atom corresponding to the pyridyl ring [38]. However, the bands at 1637 cm^−1^ and 1200 cm^−1^ remain unshifted. 

The size distribution of the gold and silver nanoparticles was evaluated. The analysis was conducted to verify how the irradiation time during the laser ablation synthesis affected the average size of the NPs. The results are shown in Table 1. The average particle size distributions of Au and Ag NPs synthesized by different ablation times are shown. The particles at 5 min are larger (96 nm) than those obtained at 20 min. As the ablation time increases from 5 to 20 min, the size distribution experiences a significant decrease of NP size and the average particle size is reduced to 75 nm. Similar results were obtained by Baladi [39] in the synthesis of Al nanoparticles [40,41].

**Table 1:** Average size of Au and Ag NPs synthesized by laser ablation.

Nanoparticle Type	Irradiation Time (min)	Average Size (Z size, nm)
Au	5	96
Au	10	82
Au	15	71
Au	20	75
Ag	5	77
Ag	20	66

**Figure 4:**
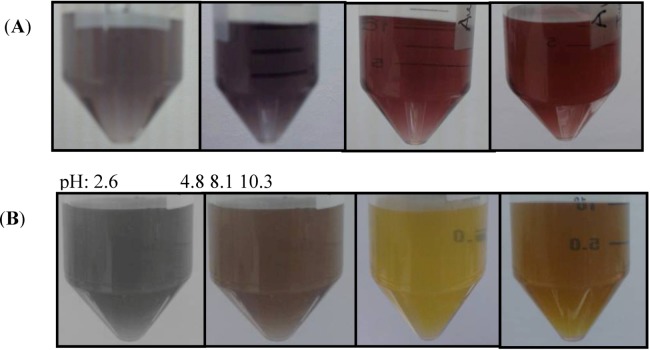
NPs suspensions at different pH values of the solvent during synthesis: (**A**) Au NPs suspensions; (**B**) Ag NPs suspensions.

The pH plays a very important role in the properties of the NPs [42] prepared, including their SERS activity [43]. A change in the ionic strength in the medium leads to the formation of clusters of particles or even a monolayer of particles on a surface. This conglomeration of NPs leads to changes in their color analogous to the variation in color associated with altering the size or the shape of the particles. When two or more particles stick together, the absorption produced is very similar to that of a single rod-like particle with a larger length [44]. Water at different pH values was used to evaluate the SERS activity of the NPs. The pH of the water was adjusted to 2.6, 4.8, 8.1 and 10.3. The irradiation time used for the syntheses was 20 min. The colors of the NPs in water depend on the pH value. Figure 4 contains color micrographs of Au and Ag NP suspensions synthesized at the various pH values studied. Differences in the predominant colloidal color were found. These color changes can be associated with the average size and the predominant shapes of the NPs in the suspensions.

**Figure 5:**
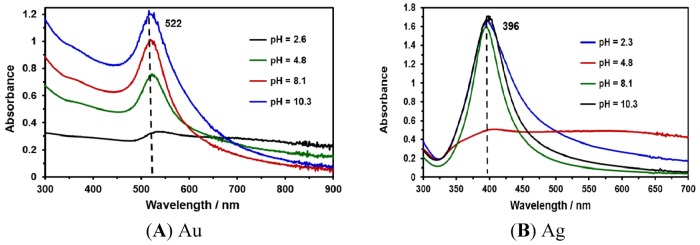
UV-Vis absorption spectra of NPs colloids after synthesis at various pH: (**A**) Au; (**B**) Ag.

Figure 5 shows the UV-Vis absorption spectra for Au and Ag nanoparticles synthesized at different pH values. A broad absorption band indicates the presence of different sizes coexisting in the colloidal suspension. At acidic pH values, a low absorbance was found for both Au and Ag NP suspensions. Therefore, at pH values of 8.1 (slightly basic) and 10.3 (basic), intense absorbances were exhibited by the suspensions. No changes in the characteristic wavelength location of the plasmon resonance absorption bands were detected for the metallic NPs under study. 

**Figure 6:**
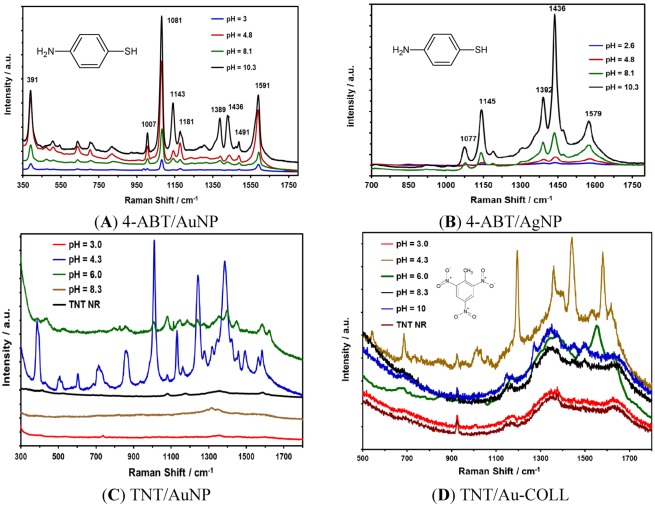
pH effect on SERS activity: (**A**) SERS spectra of 4-ABT on the Au NPs/Au substrate; (**B**) SERS spectra of 4-ABT on the Ag NPs/Au substrate; (**C**) TNT SERS spectra on the Au NPs/Au substrate; and (**D**) SERS spectra of TNT interacting with a Au colloidal suspension, included for comparison.

Au or Ag NPs suspensions at different pH values were transferred onto gold-coated glass slides to prepare the SERS substrates. Then, the samples were allowed to dry, and 5.0 μL of 1 mM 4-ABT was deposited on the substrates. The SERS measurements were acquired using a Raman excitation source at 532 nm for the analytes deposited on Ag NPs and 785 nm for the Au NPs substrates. The results for 4-ABT on Au and Ag NPs are shown in Figure 6.

The SERS spectra of 4-ABT on Au NPs shown in Figure 6A are in good agreement with previous results [32]. The four strong peaks at 1591, 1436, 1389 and 1143 cm^−1^ can be assigned to ring 8b, 19b, 3 and 9b modes of 4-ABT, respectively. The peaks at Raman shifts of 1491 and 1081 cm^−1^ are due to 8a and 19a modes, respectively, that possess the a1-type of symmetry [26,45]. For Au NPs, the best results were found at pH values of 10.3 and 4.8. Kim and collaborators found that the b2-type bands of 4-ABT are strongly affected by the solution pH [33]. Regardless of the excitation wavelength and the type of SERS substrates, the b2-type bands appeared very weak or negligible at acidic pH, while they were observed very distinctly at basic pH in Ag NPs. Our results show differences in the signals at 1143 and 1181 cm^−1^ for Au NPs at different pH values. Kim [46] attributes the disappearance of the b2-type bands at acidic pH to the protonation of the amine group, thus causing the charge transfer resonance chemical enhancement to be less likely to occur.

Likewise, the results for 4-ABT obtained for Ag NPs deposited on Au-coated glass slides presented good enhancement in the Raman signals, specifically the NP suspensions prepared at basic pH. Figure 6B shows the Raman signals for 1 mM 4-ABT. To determine the surface enhancement factor (SEF) for 4-ABT, aliquots of 1.0 × 10^−9^ M were deposited on the NP substrates. The deposited sample covered an area of approximately of 0.25 cm^2^. If the spot of the laser using an objective of 10× is approximately 13 μm^2^, the number of 4-ABT molecules that were illuminated was calculated to be 1.4 × 10^5^, which represents a SEF of 1.6 × 10^9^. 

To determine the effect of pH on Au NPs in SERS activity for the detection of TNT, 3 µL of a solution of the explosive at 1.17 mM was deposited on different Au substrates with gold NPs deposited. The NPs were synthesized at various pH values. The best results were observed at a pH value of nanoparticles of 4.3 and 6.0. The particles obtained at acidic pH (~3) were very unstable for Au NPs. The NPs were precipitated approximately 1 h after preparation. Colloidal results are shown in Figure 6D to compare the results of nanoparticles deposited versus colloidal suspensions of nanoparticles. A poor enhancement is observed in the colloidal suspension at pH 4.3. The results confirm one of the intrinsic limitations of metal colloidal nanoparticles for SERS applications, where their robustness as SERS substrates is compromised. An important factor in the enhancement obtained through SERS is the need for aggregation to generate the necessary plasmonic conditions for the production of significant SERS. If adequate aggregation does not occur, the reproducibility of SERS on colloidal NPs is affected because the kinetics of the process can be uncontrollable once aggregation begins.

**Figure 7:**
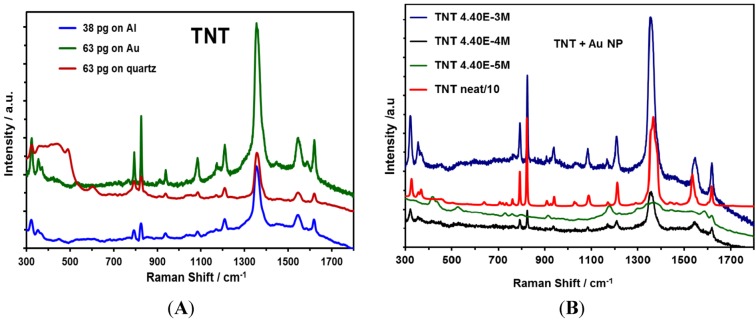
SERS spectra of TNT deposited on Au NPs; spectra were taken at 785 nm: (**A**) NPs were deposited on different substrates (Al film, Au film and quartz); (**B**) TNT deposited at different concentrations and Al was used as the substrate.

SERS is an important technique to develop applications for the detection of highly energetic materials (HEM) [47,48,49,50,51,52,53,54]. TNT is a HEM of vast military applications and uses. TNT was selected as a nitroaromatic HEM to evaluate Au NPs on different substrates. Solutions containing TNT were transferred onto various substrates containing Au NPs that were deposited and immobilized. Substrates, such as Al and quartz plates, were also used to deposit Au NPs. TNT solutions at different concentrations (1.0 × 10^−4^ M, 1.0 × 10^−6^ M and 1.0 × 10^−10^ M) were used to determine the lowest amount of HEM that could be quantitatively detected (Low Limit of Detection, LOD). The results shown in Figure 7 indicate an increase in the intensity of the principal vibrational signals of TNT due to SERS, which presents strong bands. 

The signal observed corresponded to the presence of the NO_2_ group out-of-plane bending mode at 826 cm^−1^ and the NO_2_ stretching mode at 1300–1370 cm^−1^ [55]. Primera-Pedrozo *et al.* reported similar results in 2008 [31], where the enhancement was obtained from colloidal gold NPs with a modification of the ionic strength of the media that was used to interact with the explosive. A SEF of 2 × 10^9^ was obtained for TNT.

The zeta potential of a system is a measure of the charge stability and controls all particle-particle interactions within a suspension. Understanding the zeta potential is of critical importance in controlling the dispersion and determining the stability of a nanoparticle suspension, *i.e.*, to what degree aggregation will occur over time [56]. A lower level of the zeta potential results (0 to ±30 mV) in a smaller electrostatic repulsion between the particles, maximizing aggregation/flocculation. Zeta potential measurements of the as-prepared samples yielded values of −34.9 mV for the Au colloid and −20.9 mV for the Ag colloid, thus confirming the moderate stability of gold and silver nanoparticles. Similarly, the Z-potential and the Z-size were acquired for colloidal suspensions of Ag and Au NPs at different pH values. The results confirm the color differences in the colloidal suspension when the pH was adjusted to the solutions before the synthesis. The results are shown in Table 2. The values of the Z potential reflect that the stability of the Au NPs is compromised at different pH values of the synthesis of colloidal suspensions. For this reason, the suspended colloidal NPs result in low reproducibility, lower SEF values and higher LODs.

**Table 2:** Results of the Z-potential and the Z-size for Au NPs and Ag NPs at various pH values.

NP Type	pH	Average Size (nm)	Z Potential (mV)
Ag	4.2	96	−18.0
Ag	6.0	90	−21.8
Ag	8.3	87	−24.1
Ag	10.0	72	−26.2
Ag	10.8	70	−29.1
Au	4.3	93	−11.1
Au	6.0	75	−39.2
Au	8.3	73	−3.7
Au	10.0	47	−5.2

Moreover, we have determined the number of TNT molecules that were present on the Au NPs for an area of approximately of 1.3 × 10^7^ µm^2^ deposited on a substrate. The interrogation area (circular) or laser spot when using a 20× objective was 2.3 × 10^3^ µm^2^. The TNT mass contained in the interrogated area for the highest concentration solution was 7.8 × 10^−12^ g. The number of TNT molecules that were SERS excited was calculated to be 2.1 × 10^10^ molecules (1.0 × 10^−4^ M, 2 µL deposited), 2.1 × 10^8^ molecules (1.0 × 10^−6^ M, 2 µL) and 2.1 × 10^4^ molecules (1.0 × 10^−10^ M, 2 µL).

The data were compared with experiments using colloidal NPs. The Raman signals were not observed in colloidal NPs. A possible explanation is that the acquisition of good enhanced Raman signals depends on the reliability and stability of the SERS-active sites (or “hot spots”), which have a large influence on the enhancement of the Raman signal intensities. However, the enhancement in the signals obtained depends significantly on the aggregation of Ag or Au colloids and the analyte used in the analysis. The stability of metal colloids is due to the repulsive forces derived from the charged species on the surface of the colloidal particles, which assume a nonzero effective charge. When these charges are replaced with a neutral adsorbate, aggregation occurs, usually when a ligand has a greater affinity for the metal than that of the surface charged species [57].

## 4. Conclusions

Colloidal suspensions of Ag and Au NPs were successfully synthesized by laser ablation using water as the solvent. Colloids of different colors and sizes were obtained, depending on the time of irradiation in the synthesis and the pH of water. Excellent SERS results were found for Au and Ag NPs deposited on Au films using 4-ABT at a pH value of 10.3.

The potential application of Au and Ag nanoparticles in SERS detection of explosives was evaluated. We have detected 7.8 × 10^−^^18^ g of TNT on Au NPs deposited on Al sheets. The SEF obtained establishes the possibility of using the substrates prepared for the detection of contaminants in water, such as nitroaromatic HEM.
